# Developing and Adapting a Family-based Diabetes Program at the U.S.-Mexico Border^1^

**Published:** 2004-12-15

**Authors:** Nicolette I Teufel-Shone, Rebecca Drummond, Ulrike Rawiel

**Affiliations:** Mel and Enid Zuckerman Arizona College of Public Health; Mel and Enid Zuckerman Arizona College of Public Health, University of Arizona, Tucson, Ariz; Mel and Enid Zuckerman Arizona College of Public Health, University of Arizona, Tucson, Ariz

## Abstract

**Context:**

The prevalence of diabetes among Hispanics is more than twice that of non-Hispanic whites in communities along the U.S.-Mexico border. The University of Arizona and two community health agencies on the Arizona border, *Campesinos Sin Fronteras* and Mariposa Community Health Center, collaborated to design, pilot and assess the feasibility of a lay health-outreach worker- (*promotora-)* delivered diabetes education program for families. *La Diabetes y La Unión Familiar* was developed to build family support for patients with diabetes and to teach primary prevention behaviors to family members.

**Method:**

Community and university partners designed a culturally appropriate program addressing family food choices and physical activity, behavior change, communication, and support behaviors. The program offers educational content and activities that can be presented in home visits or multifamily group sessions. Community partners led the implementation, and university partners guided the evaluation.

**Consequences:**

Seventy-two families (249 total participants) including children and grandchildren participated. Preintervention and postintervention questionnaires completed by adults (n = 116) indicate a significant increase in knowledge of eight diabetes risk factors (*P* values for eight factors range from <.001 to .006) and a significant increase in family efficacy to change food (*P* < .001) and activity behaviors (*P* < .001). Interviews with participants highlight the program's positive psychosocial impact.

**Interpretation:**

Community and university collaboration involved building upon the*promotoras'* expertise in engaging the community and the university's expertise in program design and evaluation. A *promotora*-delivered family-based diabetes prevention program that emphasizes family support, communication, and health behaviors is feasible and can yield change in family knowledge, attitude, and behavior relative to diabetes risk factors.

## Background

Among Hispanic populations in the United States, rates of type 2 diabetes and secondary complications (e.g., retinopathy, neuropathy, renal failure) are more than twice those reported for non-Hispanic whites (1-3). The high prevalence of associated conditions corresponds to the poor rates of adherence to American Diabetes Association recommendations for diabetes self-management documented for this population (4,5). Hispanic adults in the United States with diabetes and cardiovascular disease cite attitudes, perceptions, and preferences of family members as significant barriers to making recommended changes in their diet and exercise patterns (4,6,7). Yet family members of Hispanic patients with diabetes are at a particularly high risk for developing the disease themselves because of their family history of diabetes and because of high rates of risk factors (e.g., gestational diabetes, obesity, physical inactivity) in this population (1,8). Providing diabetes education to the entire family would address both prevention and treatment.

Because of a strong cultural emphasis on family connectedness among Hispanics, family members may be particularly influential in the development of health behaviors in this population (7,9-11). Family behaviors and attitudes can support or challenge a patient's psychosocial adaptation to illness and subsequently a patient's confidence, intent, and willingness to implement disease-management strategies (12-15). In a study of 76 Hispanic patients with diabetes, Chesla et al (16) reported that 23% of the patients noted troubling changes in family relations since their diagnosis, and 32% feared that more changes created by the social and financial stress of the patient's increasing dependency on other family members were imminent.

In several studies from Mexico, family support has been shown to have significant positive association with glucose control in patients with diabetes (17,18). Family diabetes education studies in Cuba (19) and Costa Rica (20) have demonstrated improved glycemic control and treatment compliance in patients whose families had information about diabetes and were supportive of recommended health behaviors. In the United States, family education and health-behavior change among Hispanic populations has been related to weight loss. Foreyt et al (21) and Cousins et al (22) demonstrated that interactive family-oriented weight-loss interventions yield greater success than approaches that target the individual. Both studies highlight the importance of family support within Hispanic culture and call for additional efforts to evaluate the effectiveness of family-oriented health interventions.

Despite the documentation of family influence on health behaviors, particularly within Hispanic populations, the literature offers no U.S. examples of diabetes interventions that target the family and address the family's collective behaviors. Brown et al (23,24) encourage the involvement of a family member in support groups in a diabetes self-management intervention but do not specifically target the family or family behaviors.

*La Diabetes y La Unión Familiar* (Diabetes and the Family) is the family component of the Border Health Strategic Initiative (*Border Health ¡SI!*). *Border Health ¡SI!* is a comprehensive community-based diabetes prevention and control project developed by the Mel and Enid Zuckerman College of Public Health at the University of Arizona (MEZACOPH) and various community partners serving Arizona residents at the U.S.-Mexico Border. The various components and multiple community-university partnerships of *Border Health ¡SI!* are described in this issue (25).

The objectives of this family-based diabetes education intervention are the following: 1) to enhance family members' social support of patients with diabetes and 2) to increase the range of primary prevention behaviors associated with diabetes in family members of patients with diabetes. This community case study describes the collaborative development, delivery, and outcome of the initial implementation of *La Diabetes y La Unión Familiar*.

## Context

Geographically and demographically, the context of *Border Health ¡SI!* and the family component are the underserved Hispanic communities of Yuma and Santa Cruz counties, located along Arizona's southern border with Mexico. Both counties report an annual median household income of less than $33,000 with greater than 19% of residents living below the poverty level (26). In addition, 43.5% of households in Yuma County and 79.2% in Santa Cruz County report that Spanish is the predominant language spoken at home (26). Many residents in these counties do not seek regular health care because they lack access to Spanish-speaking health care providers and do not have access to health insurance (20% of Arizona residents do not have health insurance); some are fearful about their immigration status (26,27).

To adapt to the needs of Hispanic families in these counties and to the skills of local *promotoras,* MEZACOPH, *Campesinos Sin Fronteras* (*CSF*) in Yuma County, and Mariposa Community Health Center (MCHC) in Santa Cruz County collaborated to develop, implement, and evaluate *La Diabetes y La Unión*. The *promotoras* from MCHC had extensive experience in providing education and support to Hispanic patients with diabetes. The *promotoras* from *CSF* had received national attention for their health education efforts extended to Hispanic families and migrant farm workers, but they had limited experience with diabetes education. In both communities, the *promotoras* reported the need for a diabetes intervention that addressed family support and education. Program development used *promotoras'* knowledge of local context and experience in providing outreach services to this population.

## Methods

### Development

MEZACOPH investigators, *CSF* and MCHC directors and *promotoras*, and the MCHC-certified diabetes educator met in a day-long work session to discuss local strengths, challenges, and educational needs. The group reached consensus on module subject areas, order, format, appropriate instructional styles, and community differences that might influence recruitment, retention, and program delivery. After this initial meeting, MEZACOPH investigators met separately with *promotoras* from each site in monthly meetings over an eight-month period to review draft instructional materials and to gain feedback on the approach, format, and translation of the developing curriculum. In these meetings, *promotoras* from both sites also provided examples of health education materials that had been well received in their communities.

### Training

Once the curriculum was developed, a bilingual MEZACOPH investigator conducted a day-long training session in Spanish with *CSF* and MCHC *promotoras.* Since the *promotoras* had contributed to the development of many of the materials incorporated into the curriculum, the training offered an introduction to the overall flow and format of the curriculum and instruction in the use of educational materials. *Promotoras* gained familiarity and comfort with the curriculum during and after the training through playing roles, practicing delivery, and having coworkers critique their style.

### Intervention

*La Diabetes y La Unión Familiar* is a 12-week program with 10 points of contact: three home visits, five educational sessions, and two celebratory events. Drawing on key concepts of Social Learning Theory, a theoretical model was developed to guide the program design process (Figure). Key concepts include the influence of the social environment in behavior change, the need for knowledge and skills to change behavior (behavioral capability), and the importance of building confidence in the ability to take action (self-efficacy). Intervention activities include teaching team-building and communication skills to build and reinforce intrafamily communication, collective esteem (so all family members accept and value the family as a group), and collective efficacy (which promotes confidence in the family's ability to make changes). Intervention activities also include providing information on food choices and physical activity so families can make informed choices. Key concepts related to diet, exercise, and family support are introduced and discussed through the use of pictorial flipcharts, educational games, food sampling and preparation, and low-level physical activities.

Family social behaviors such as cohesion, adaptation (exhibited by resiliency and problem solving), and support within the family influence family food choice and physical activity behaviors. The proposed outcome of targeting family social behaviors to change health behaviors is improved nutrient intake, activity level, and diabetes management or prevention for all family members. The program encourages family members to collectively set health-behavior goals, to overcome obstacles hindering healthy behaviors, and to develop a plan to sustain behavior changes.

**Figure:**
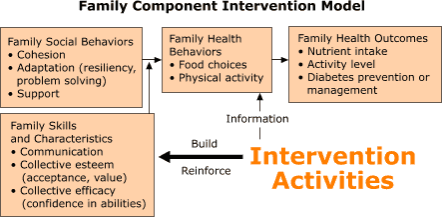
A family-based diabetes control and prevention program at the U.S.-Mexico border.

**Figure. **A family-based diabetes control and prevention program at the U.S.-Mexico border.

The *promotora* instructional manual provides an overview of the goals and format of the program, a description of the objectives, and an outline of the activities and supplies needed for each point of contact. Each family is given a notebook that includes copies of the flipchart materials and pockets for handouts, recipes, and other memorabilia such as photographs and a graduation certificate.

To accommodate differences in *promotora* skills and community characteristics, curriculum delivery is flexible. Educational flipcharts and games are prescribed for specific modules, but foods/snacks, exercises, and supplemental activities (e.g., additional games and stories) are selected by the *promotoras* from appendices in the instructional manual.

The program sequence and content are described below:

- *Conocer a la Familia* (Meet the Family) is the first home visit to explain the length and format of the program to the family, register interested family members (name, age and relationship to the family member with diabetes), gain informed consent for participation in evaluation activities, and administer a preintervention questionnaire to all participating family members aged 18 years and older. All consent and evaluation procedures were approved by the University of Arizona Institutional Review Board.
- *Bienvenidos!* (Welcome!) is a kick-off event for all families.
- Five weekly interactive educational modules are described below:
  - *Familias y Diabetes* (Families and Diabetes) is a general introduction addressing diabetes risk factors, symptoms, and complications.
  - *Ser Saludable* (Being Healthy) addresses the relationship between physical activity, food choices, and diabetes control and prevention.
  - *Crear Metas* (Creating Goals) asks families to evaluate their own current health behaviors and provides steps for creating, reaching, and maintaining health-behavior goals.
  - *La Unión Familiar* (Working Together) encourages families to discuss their progress and goals and suggests ways they can enhance their success through family support and unity. This module expands the discussion of support by teaching all family members to recognize, avoid, and remedy low and high blood sugar.
  - *Seguir Saludable* (Staying Healthy) encourages families to continue to support one another in reaching goals and to create new goals as previous goals are obtained. In this module, family communication and support skills useful in recognizing and coping with the stress and depression that often accompany diabetes are discussed.
- *Felicidades!* (Congratulations!) is a celebratory event for all families to acknowledge completion of educational modules.
- *Cómo Están?* (How Are You?) is the second home visit and provides an opportunity for *promotoras* to meet with each family to discuss progress and challenges to family health goals.
- *Evaluación* (Evaluation) is the third and final home visit and provides an opportunity for an outside evaluator to administer the postintervention questionnaire to all participating family members aged 18 years and older.

All training, intervention, and evaluation materials and activities were produced and delivered in Spanish.

#### Recruitment

Two *CSF* and two MCHC *promotoras* implemented the program at their respective sites. *Promotoras* contacted patients who had completed the *Border Health ¡SI!* patient education classes and extended an invitation to participate in a family diabetes education program. If a patient expressed interest, one or two *promotoras* made a home visit, scheduling a time when interested family members might be present. See previous description of this first meeting under*Conocer a la Familia *(Meet the Family).

#### The term

*family* was defined by the patient and included spouses, children, parents, siblings, and friends. Even family members and friends not living in the same household with the patient could be identified as family if they had weekly contact with the patient. To avoid barriers created by child-care needs, no age limit was imposed, and even infants were permitted to attend. In the group-delivery format, child care was provided. Children aged less than 18 years participated in the intervention but were not included in assessment activities. Once registered, all family members were invited to the kick-off event (*Bienvenidos!*) to meet other families participating in the intervention to play games and to enjoy a healthy meal.

#### Delivery

Each agency chose a different delivery style for the five educational modules based on their experience with family education efforts in their communities. In Yuma County, two *promotoras* delivered the five sessions through a series of weekly home visits. In Santa Cruz County, two *promotoras* delivered the modules in weekly evening classes to five to 10 families in a group format at a central location; if requested during the initial home visit, *promotoras* provided transportation and child care. Home visits and celebratory events were implemented comparably at the two sites.

#### Retention

In the group format, families who missed an education session were called or visited by a *promotora* to determine if transportation, illness, lack of interest, or other barriers had prevented their attendance. The *promotora* would offer to help solve any problem, such as providing transportation or reassuring participants who felt uncomfortable with the format or information. In the home delivery, retention was not an issue. Families were consistently home at the prearranged time.

### Assessment

Impact of the intervention was assessed using the following: 1) a written preintervention and postintervention Knowledge, Attitudes, Beliefs, and Behaviors (KABB) questionnaire with 15 close-ended items that documented adult participants' self-reported knowledge of risk factors, dietary and exercise habits, perception of need to eat healthy foods and be active, collective (family) exercise habits, and collective efficacy to make behavior changes; and 2) postgraduation interviews. Questions were designed to track changes in the stated learning objectives of each of the educational sessions. *Promotoras* reviewed the questionnaires for readability and comprehension. A team of university *Border Health ¡SI!* investigators reviewed the individual questions and entire questionnaire for content and face validity. The design of the questionnaire was driven by community and university collaborators' intent to develop a user- and administrator-friendly instrument that the *promotoras* would continue to use to document program impact.

The McNemar test for paired categorical data and the Wilcoxon signed rank test for paired continuous data were used to compare preintervention and postintervention responses of 116 adult participants (48 Santa Cruz County and 68 Yuma County).

In the initial visit, *promotoras* administered the preintervention questionnaire. In the final home visit, an evaluator not identified with the intervention administered the postintervention questionnaire. To accommodate variations in literacy levels and to avoid bias, all administrators were instructed to use a straightforward objective style when reading questionnaires aloud in Spanish. Participants were instructed to make their selection independently.

In the second home visit (*Cómo Están?) *) two weeks after graduation, *promotoras* were impressed by participants' comments emphasizing the psychosocial importance of the program. To further explore an outcome not assessed by the preintervention and postintervention questionnaire, MEZACOPH investigators decided to conduct follow-up interviews with a sample of participants. One year after graduation, a sample of 18 participants consented to an open-ended interview with a MEZACOPH investigator not identified with the intervention. Given the small sample and an interest in documenting all experiences within this case study, participants' statements, as recorded in writing by the interviewer, were grouped by common themes.

## Consequences

The following outcomes of the initial implementation of *La Diabetes y La Unión Familiar* are offered in support of the feasibility and potential impact of a *promotora-*delivered family-based diabetes education intervention.

### Participation

Four rounds of *La Diabetes y La Unión Familiar* were implemented in each intervention county, yielding 72 patients with diabetes and 177 support people, including children and grandchildren. Table 1 illustrates the distribution of participants by sex and age group; figures include all patients with diabetes, family members, and supporters. Twenty-five percent of participants were children (younger than 18 years). Depending on their age and interest, children participated in the games, listened to stories, and participated in discussions generated by the flipcharts. Younger children and infants played or were cared for in a separate child-care area in the multifamily group sessions.

Adult daughters and wives were the predominant participating supporters. Percentage of supporters by relationship to the family member with diabetes was: 22% daughters, 20% spouses (with 54% wives and 46% husbands), 15% sons, and 9% friends. Of the total adult participants, 87% attended three or more of the five educational modules, and 43% of the youth and adults attended eight or more of the 10 points of contact.

### Preintervention and Postintervention Knowledge, Attitudes, Beliefs, and Behaviors (KABB) Outcomes

Sixty-one (53% of total participants) of the preintervention and postintervention response pairs were from the family member with diabetes.

Table 2 provides the percent of yes responses to a list of possible diabetes risk factors listed in the questionnaire. A family history of diabetes (heredity) and being Hispanic, overweight, inactive, and older than 45 years were introduced in the first educational session (*Familias y Diabetes*) as known risk factors for diabetes. Based on their outreach experience, *promotoras* reported that some clients believed that stress, fear, and contact with an individual with diabetes were also risk factors. This first session provided an opportunity to discuss these perceptions. Preintervention and postintervention test comparisons indicate a significant increase in participants who identified the five known risk factors and a significant decrease in those who indicated yes to stress, fear, and contact after the intervention.

Table 3 provides a comparison of participants' preintervention and postintervention responses to family efficacy questions. Participants responded to the following questions on a five-point scale with 1 = not very confident and 5 = very confident: How confident are you that your family can become more physically active? How confident are you that your family can eat healthier? After the intervention, participants report a significant increase in their perception of their family's efficacy to make specific behavior changes.

Questions of food intake and activity were changed during the course of the program in response to questionnaire administrators' report that participants may be misinterpreting questions. The initial questions asked about general intake of specific foods and participation in specific activities. Questionnaire administrators indicated that respondents did not believe that they had regular food and activity behaviors and answered based on their behavior over the previous week. To create a questionnaire responsive to the administrators' observations and to provide an evaluation instrument that allowed participants to report behavior change in their own terms, these questions were changed after two rounds of administration from "in general" to "in the last week." Given the change, responses to these two different sets of questions were analyzed separately (separate data table not provided). Despite the difference in question wording, these separate data sets do reflect similar patterns of change, which are described below:

- The frequency of sweetened drink consumption decreased significantly (*P* < .001 for both response to question on general intake as well as question on previous week). These drinks included fruit drinks distinctive within Hispanic culture (e.g. horchata, tamarindo, jamaica, Tampico™ as well as Gatorade™ and Sunny Delight™, but not carbonated soft drinks.
- No consistent change was noted in reported fruits, vegetables, soft drinks, or low- and nonfat milk consumption.
- A nonsignificant trend in respondents reportedly exercising five or more times per week for 30 minutes or more.
- A significant increase in family members participating together in a physical activity (*P* = .002).
- A significant increase in participants reporting that family members help and support each other (*P* = .01).
- A nonsignificant trend toward greater communication and cohesive behaviors, such as talking about food choices, going to the doctor with the family member with diabetes, and agreeing to eat out or buy food from places with healthy choices.

Changes in knowledge, attitudes, behaviors, and beliefs were not different in a comparison of family members with diabetes and family members without diabetes.

### Interviews

Eighteen individuals (both patients and family members) from both sites were interviewed individually for approximately one hour. All statements could be grouped into one of three themes:

- Program participation had a positive psychosocial impact on participants. Those with diabetes and family members reported feeling better and being less depressed and isolated.
- Family communication, particularly about food choices and understanding of depression, improved; communication was more frequent and/or less emotional.
- The social interaction provided by the *promotoras* was the best part of the program.

No other follow-up data were collected to assess long-term behavior change or retention of knowledge.

## Interpretation

*La Diabetes y La Unión Familiar*, a Spanish language family diabetes education intervention that targets family support, communication, and family health behaviors, implemented by *promotoras* in two Arizona border communities, yielded changes in family members' knowledge, attitudes, behaviors, and beliefs relative to diabetes prevention and control.

The development of the program content, delivery format, and even evaluation methods was a collaborative process among a university, MEZACOPH, and two community health agencies, *CSF* and MCHC. The description of the collaborative process illustrates how standard research practices and community experience, observation, and interests contributed to the final intervention. Program outcomes demonstrate that teaching the family as a group can influence health behaviors, yielding an increase in family-based physical activity and ameliorating family member feelings of depression and isolation. This community case study supports the use of a family-based approach to diabetes prevention and control. This study indicates that family involvement should go beyond diabetes support groups that tend to focus only on the behaviors of the person with diabetes. Addressing the family's collective behaviors as well as patterns of cohesion and communication can yield change in the family environment, an important influence in chronic disease management and prevention.

Certain preexisting factors and limiting conditions of this case study should be acknowledged. The *promotoras* had previous experience and training in community outreach services. They were uniquely familiar with the curriculum as they collaborated in its development and adapted its delivery for their communities' needs. The small sample size in this case study limits the authors' ability to project the applicability and impact of *La Diabetes y La Unión Familiar* in other communities. Yet these results are promising and warrant continued implementation of the program in these counties and piloting in similar communities. The program is available to other agencies by accessing its Web site (available from: http://www.borderhealthsi.org/). Furthermore, the preintervention and postintervention evaluation instrument did not capture the psychosocial impact of the program as revealed by *promotoras'* observations and a small number of interviews. Future implementation should consider revising the evaluation instrument or supplementing evaluation activities with a formal guided interview conducted by an evaluator not identified with the intervention.

## Figures and Tables

**Table 1:** Participation in Family-based Diabetes Program From Two Intervention Sites in Yuma and Santa Cruz Counties, Arizona

	**Male**	**Female**	**Total**
**Adults (≥18 years)**	49 (27%)	135 (73%)	184 (75%)
**Youth (<18 years)**	36 (55%)	29 (45%)	65 (25%)
**Total**	85 (34%)	164 (66%)	249 (100%)

**Table 2:** Adult Participants’ (N = 116) Preintervention and Postintervention Responses to the Knowledge, Attitude, Beliefs, and Behaviors (KABB) Questionnaire: Knowledge of Risk Factors, Yuma and Santa Cruz Counties, Arizona

	Yes n (%)		
		
**Diabetes risk factors^a^**	**Preintervention**	**Postintervention**	***P* b**
Heredity (family history)	86 (74.1)	104 (89.7)	.002
Hispanic	39 (33.6)	94 (81.0)	<.001
Overweight	85 (73.3)	106 (91.4)	.001
Inactive	69 (59.5)	109 (94.0)	<.001
>45 years of age	51 (44.0)	102 (87.9)	<.001
Stress	68 (58.6)	46 (39.7)	.002
Fear	81 (61.8)	50 (38.2)	<.001
Contact	3 (75.0)	1 (0.9)	.006

a Participants were asked to answer yes if they believed that the characteristic put them at greater risk for diabetes and to answer no if they did not believe that the characteristic put them at greater risk.

b Determined by two-tailed test.

**Table 3:** Adult Participants’ (N = 116) Preintervention and Postintervention Responses to the Knowledge, Attitude, Beliefs, and Behaviors (KABB) Questionnaire: Family Efficacy, Yuma and Santa Cruz Counties, Arizona

**Confidence in ability of family to change behavior**	**Mean ± SD**		***P* a**
		
	**Preintervention**	**Postintervention**	
To eat healthier foods	3.42 ± 1.22	4.13 ± 1.19	<.001
To be more physically active	3.46 ± 1.20	4.00 ± 1.20	<.001

a Determined by two-tailed test.
